# DNA extraction protocol impacts ocular surface microbiome profile

**DOI:** 10.3389/fmicb.2023.1128917

**Published:** 2023-04-20

**Authors:** Heleen Delbeke, Ingele Casteels, Marie Joossens

**Affiliations:** ^1^Department of Ophthalmology, University Hospitals Leuven, Leuven, Belgium; ^2^Department of Neurosciences, Research Group Ophthalmology, Biomedical Sciences Group, KU Leuven, Leuven, Belgium; ^3^Laboratory of Microbiology, Department of Biochemistry and Microbiology (WE10), Ghent University, Ghent, Belgium

**Keywords:** DNA extraction protocol, 16SrRNA sequencing, eye microbiome, ocular surface microbiome, sequencing results

## Abstract

**Purpose:**

The aim of this study is to provide a reference frame to allow the comparison and interpretation of currently published studies on 16S ribosomal ribonucleic acid amplicon sequencing of ocular microbiome samples using different DNA extraction protocols. Alongside, the quantitative and qualitative yield and the reproducibility of different protocols has been assessed.

**Methods:**

Both eyes of 7 eligible volunteers were sampled. Five commercially available DNA extraction protocols were selected based on previous publications in the field of the ocular surface microbiome and 2 host DNA depletion protocols were added based on their reported effective host DNA depletion without significant reduction in bacterial DNA concentration. The V3-V4 region of the 16S rRNA gene was targeted using Illumina MiSeq sequencing. The DADA2 pipeline in R was used to perform the bio-informatic processing and taxonomical assignment was done using the SILVA v132 database. The Vegdist function was used to calculate Bray-Curtis distances and the Galaxy web application was used to identify potential metagenomic biomarkers *via* linear discriminant analysis Effect Size (LEfSe). The R package Decontam was applied to control for potential contaminants.

**Results:**

Samples analysed with PowerSoil, RNeasy and NucleoSpin had the highest DNA yield. The host DNA depletion kits showed a very low microbial DNA yield; and these samples were pooled per kit before sequencing. Despite pooling, 1 of both failed to construct a library.

Looking at the beta-diversity, clear microbial compositional differences - dependent on the extraction protocol used – were observed and remained present after decontamination. Eighteen genera were consistently retrieved from the ocular surface of every volunteer by all non-pooled extraction kits and a comprehensive list of differentially abundant bacteria per extraction method was generated using LefSe analysis.

**Conclusion:**

High-quality papers have been published in the field of the ocular surface microbiome but consensus on the importance of the extraction protocol used are lacking. Potential contaminants and discriminative genera per extraction protocol used, were introduced and a reference frame was built to facilitate both the interpretation of currently published papers and to ease future choice – making based on the research question at hand.

## Introduction

The increasing interest in the link between microorganisms and human health resulted in several high-quality papers that describe the microbial composition of the ocular surface. At present, comparing or summarizing the state-of-the-art regarding the ocular surface microbiome proofs however, to be challenging due to the variety of methods used. Biases are introduced due to differently used sampling procedures, sequencing approaches, and variability in usage of topical anaesthetics, among others (Delbeke et al., 2021). However, in line with data from gut microbiota research, the biggest impact is expected to result from differences in DNA extraction protocols (Costea et al., 2017). After reporting on the immediate effect of topical anaesthetics on ocular microbiome results (Delbeke et al., 2022), we now compare different protocols for community DNA extraction. It is well-known that DNA extraction methods for complex microbial communities affect the observed species diversity downstream (Costea et al., 2017; Ahannach et al., 2021), as Gram-negative and Gram-positive bacteria have different cell wall structures. Due to their higher mechanical strength, Gram-positive bacteria are known to be more inclined to be affected by the DNA-extraction method used. Simultaneously, more aggressive DNA-extraction methods can damage the DNA from Gram-negative bacteria upon their lysis, resulting in fragmented DNA template for sequencing and subsequent lower abundance of them upon analyses (Maukonen et al., 2012).

In complex microbial samples derived from voluminous matrices like faeces, it is evident to compare different extraction protocols based on the same sample (Costea et al., 2017). The low yield derived from one ocular surface sample asked for inventive measures to accurately compare different protocols.

The aim of this study is to provide a reference frame to allow the comparison and interpretation of currently published studies on 16S rRNA amplicon sequencing of ocular surface microbiome samples using different DNA extraction protocols. Alongside, the quantitative and qualitative yield and the reproducibility of different protocols has been assessed.

## Materials and methods

### Study population

For this comparative study of seven conventional DNA extraction protocols, sampling on volunteers was approved by the Ethics Committee Research UZ / KU Leuven, Belgium in accordance with the principles of the Tenets of Helsinki. This project was registered on ClinicalTrials.gov (NCT04193774). In order to avoid a potential age-effect, the study was restricted to adult volunteers between 20 and 25 years old.

**Figure 1:**
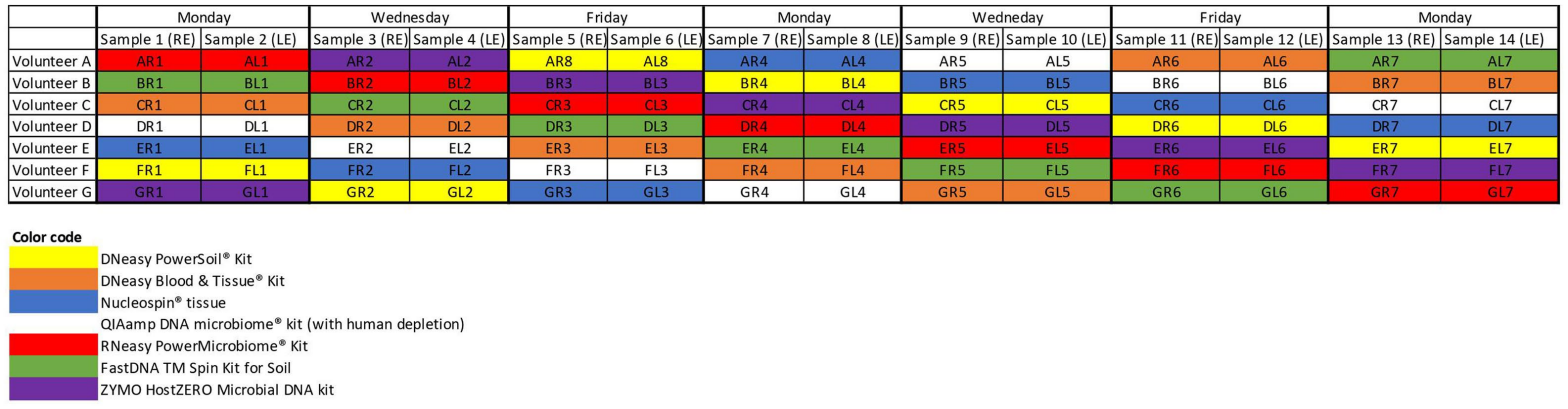
Sampling schedule. Both eyes of 7 volunteers (A–G) were samples on non-consecutive days. The colour coding matches the colour of the, respectively, used extraction protocols. RE, right eye; LE, left eye.

After giving written informed consent, all participants (*n* = 7) filled in a small questionnaire concerning potential confounding factors of the ocular surface microbiome. Samples were taken from both eyes of 7 volunteers on 7 non-consecutive days (Figure 1) (*n* = 98). Sampling was not performed on consecutive days in an attempt to reduce possible biases due to repetitive sampling. A single, sterile, nylon, flocked swab (FLOQSwabs®; Copan, Brescia, Italy) was rubbed from the nasal to temporal inferior conjunctival sac and simultaneously swirled in the opposite direction of the sampling itself. All sampling was performed by one of the authors (HD) or by a trained study nurse (IV). The swab was placed in an Eppendorf tube and immediately stored for less than 2 weeks at −18°C before being transferred to a −80°C freezer until further processing.

### DNA extraction and amplicon sequencing

Seven different commercially available DNA extraction protocols were compared (Figure 1). All samples from the same volunteer taken on a certain day were analysed with the same extraction protocol to check for repeatability and reproducibility. Samples gathered on the same day of different volunteers were further analysed with different protocols to dilute the potential impact of environmental and/or weather conditions on the microbial composition (Figure 1).

Microbial DNA was extracted from frozen samples. Commercially available DNA extraction protocols were selected based on previous publications in the field of the ocular surface microbiome: DNeasy PowerSoil kit (QIAGEN, Hilden, Germany) (PowerSoil) (Dong et al., 2011; Zhou et al., 2014; Shin et al., 2016; Cavuoto et al., 2018), DNeasy Blood and Tissue kit (QIAGEN, Hilden, Germany) (Blood & Tissue) (Doan et al., 2016; Chao et al., 2018), NucleoSpin Tissue (MACHEREY NAGEL, Dueren, Germany) (NucleoSpin) (Ozkan et al., 2017, 2018, 2019; Wang et al., 2021; Yang et al., 2022), RNeasy PowerMicrobiome Kit (QIAGEN, Hilden, Germany) (RNeasy) (Cavuoto et al., 2018), FastDNA TM Spin Kit for Soil (MP, Illkirch-Graffenstaden, France) (FastDNA) (Graham et al., 2007). Two host DNA depletion protocols were added based on their reported effective host DNA depletion without significant reduction in bacterial DNA concentration (QIAamp DNA microbiome kit (QIAGEN, Hilden, Germany) (QIAamp) (Heravi et al., 2020) and HostZERO Microbial DNA kit [ZYMO research, California, United States of America) (HostZERO)] (Ahannach et al., 2021). Host DNA depletion is especially important for metagenomic shotgun sequencing where high amounts of human DNA lead to decreased sensitivity for microbial detection (Ahannach et al., 2021). The specific adaptations made to the different extraction protocols are visualized in Supplementary 1. Every extraction protocol was used to analyse sixteen conjunctival swabs including one control and one blank.

DNA was quantified *via* fluorometry (Life Technologies Qubit dsDNA High Sensitivity Kit; Thermo Fisher Scientific, Waltham, MA). Upon low concentrations (<0.5 ng/μl), the elution buffer in the samples was partially evaporated using vacuum centrifuge (concentrator 5,301 Eppendorf) with temperatures between 30°C and 45°C until the minimal volume of 25 μl for sequencing was obtained (Supplementary 4). Library preparation and sequencing was performed at BaseClear, Leiden, The Netherlands. The hypervariable V3–V4 region of the 16S rRNA gene was amplified with PCR (341F/805R primer set). The amplified DNA was sequenced with the Illumina MiSeq sequencing platform (Illumina, San Diego, CA) to generate 2 × 300 base-pair (bp) paired-end reads.

### Data analyses

The paired-end fastq files were analysed using DADA2 pipeline version 1.12 in R (open-source version 1.2.5033) according to the tutorial for quality profiling, filtering (maximum expected error of 2) and trimming reads, sequence variants interference, removal of chimeric sequences and taxonomic assignment (R Core Team, 2014; Callahan et al., 2016). Forward reads were trimmed at 290 bp; while reverse reads were cut at the 240 bp position based on the quality profile and to maintain a minimal of 20 bp nucleotides for overlapping (Yau et al., 2019). Taxonomical assignment was done using the SILVA v132 database (Quast et al., 2013). The different extraction protocols were compared by looking at the diversity measures. Alpha diversity looks at the species diversity within a group, i.e., samples analysed with a certain extraction protocol; beta diversity looks at the species diversity between groups. Besides the number of observed species, Shannon and Simpson diversity were assessed. Both Shannon and Simpson look at the number of retrieved bacteria and their evenness of distribution. Shannon diversity is more influenced by rare species whereas Simpson diversity is more influenced by common species (Delbeke et al., 2022). Bray–Curtis dissimilarity was used to visualize beta diversity in order to examine the difference in microbial composition between the samples analysed with different extraction protocols. Alpha and beta diversity were calculated using Phyloseq (v 1.24.0). Bray-Curtis distances were calculated in R *via* vegan 2.5–7 (vegdist function). *Via* the Galaxy web application, linear discriminant analysis (LDA) Effect Size (LEfSe) (LDA threshold >2, *p* < 0.05) was executed to identify potential metagenomic biomarkers able to differentiate between the different protocols (McMurdie and Holmes, 2013; Oksanen et al., 2017; Afgan et al., 2018). LEfSe analysis was performed after the removal of chloroplasts and mitochondria. Bacteria not assigned at genus level were specified at the most precise taxonomic category assigned. LefSe was calculated using only the bacteria with a relative abundance ≥0.1% (Table 1). Extraction protocols were also grouped based on type of lyses (mechanical or chemical) (Table 2) and accompanying discriminative features were also searched for by LefSe (Supplementary 2).

**Table 1:** Discriminating genera, derived from LefSe analysis.

		RNeasy	Blood & Tissue	PowerSoil	FastDNA
**Blood & Tissue**	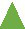	*Sulfitobacter, Pelagimonas*					
	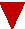	ND			
**PowerSoil**	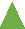	ND	*Corynebacterium_1*			
	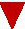	ND	*Sulfitobacter, Pelagimonas*		
**FastDNA**	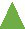	*Ralstonia*	*Ralstonia*	*Ralstonia*	
	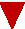	*Cutibacterium, Corynebacterium_1, Staphyloccocus, Streptococcus*	*Cutibacterium, Staphyloccocus, Streptococcus, Sulfitobacter,Pelagimonas*	*Cutibacterium, Corynebacterium_1, Staphyloccocus*	
**NucleoSpin**	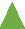	*Bacillus, Flavobacterium*	*Bacillus, Flavobacterium*	*Bacillus, Flavobacterium*	*Bacillus, Flavobacterium, Cutibacterium*
	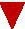	*Cutibacterium*	*Cutibacterium, Sulfitobacter,Pelagimonas*	*Cutibacterium,Corynebacterium_1*	*Ralstonia*

ND, no discriminative features, green arrow: lower abundance, red arrow: higher abundance.

**Table 2:** Type of lysis per extraction protocol.

	Chemical/Enzymatic lysis	Mechanical lysis	Heat lysis
Blood & Tissue	Proteinase K	NA	NA
NucleoSpin	Lysosyme and proteinase K	NA	2 h incubation at 56°
PowerSoil	C1	0.7 mm garnet	NA
RNeasy	PM1	0.1 mm glass beads	Additional step: 10 min at 90° (Costea et al., 2017)
FastDNA	Lysis buffer and proteinase K	1.4 mm ceramic spheres, 0.1 mm silica spheres and one 4 mm glass bead	10 min incubation at 90°

NA, not applicable.

The R package Decontam version 1.8.0 was applied separately on all samples analysed with a certain extraction protocol. The goal was to find which ASVs are most probably contaminants. We used the “frequency,” “prevalence” and “both” (frequency and prevalence combined) methods. We looked at “prevalence” with default threshold 0.1 and with threshold at 0.5 (threshold 0.5 identifies all ASVs that are more prevalent in negative controls than in the positive samples (Callahan and Davis, 2018)). The retrieved contaminants were not removed as they are of importance in this specific research project.

### Statistical analyses

The data were analysed using R 1.2.5033 statistical software (R Core Team, 2014). Normal distribution of the data was assessed using the Shapiro-Wilks normality test. When normally distributed, the mean percentage and standard deviation (SD) were used; when not normally distributed, the median percent and interquartile range (IQR) Q1–Q3 were noted. The Kruskal Wallis test was used to compare observed richness, Shannon and Simpson diversity between the different protocols. This test was followed by the Dunn Kruskal-Wallis multiple comparison test with *p*-values adjusted with the Benjamini-Hochberg method to reveal which protocols were significantly different. The Wilcoxon rank-sum test was used to compare the within-subject similarity and Bray-Curtis distances for each extraction protocol.

## Results

**Figure 2:**
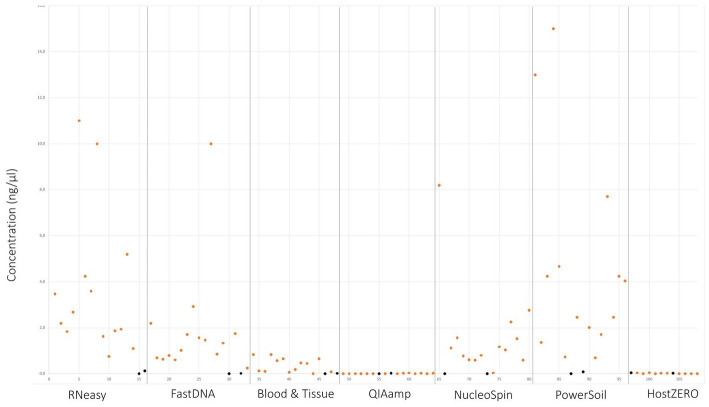
DNA yield per extraction protocol. Values in orange indicate the different sampling points. Values in black signifies controls and blank samples.

Upon written informed consent, 7 volunteers, 3 males and 4 females, participated in this study. All participants were between 22 and 25 years old and Caucasian. Two females were contact lens wearers (volunteer C and E), and two volunteers frequently rub their eyes (volunteer F and G). There was a lot of variation in sleeping position making it impossible to draw any conclusion on this confounder. Both eyes of the 7 volunteers were sampled on 7 non-consecutive days (Figure 1), signifying that 14 samples per participant were analysed. In total 98 conjunctival samples were collected. Alongside, 7 negative control swabs (unused swab opened at the sampling location and stored together with the conjunctival swabs) and 7 extraction blanks (as reagent control (Ozkan et al., 2019)) were collected, this resulted in 112 samples for analyses. Of note, the NucleoSpin blank, despite having an undetectable low amount of DNA (Figure 2), showed a high number of reads of *Bacillus* and *Flavobacterium*, both in the same order size as the other samples, upon sequencing.

### Quantitative comparison

Samples analysed with PowerSoil, RNeasy and NucleoSpin had the highest microbial DNA yield (Figure 2; Table 3; Supplementary 4). The final concentration of 36 samples was too low for downstream analyses, hence they were concentrated using the vacuum centrifuge (Supplementary 4). As all swabs analysed with QIAamp and HostZERO showed a very low microbial DNA yield, these swabs were pooled per kit before sequencing. Furthermore, only the NucleoSpin blank was sequenced as the amount of DNA was too low for sequencing in the other swabs Upon sequencing, the pooled samples analysed with the host DNA depletion kit QIAamp nevertheless failed library construction. Next to the pooled QIAamp sample, also the control sample analysed with the PowerSoil kit failed to construct a library.

**Table 3:** Overview of the different DNA extraction kits concerning microbial DNA yield, pricing, time efficiency and diversity.

	RNeasy	FastDNA	Blood & Tissue	NucleoSpin	PowerSoil	HostZERO	QIAamp
Median amount of microbial DNA (ng/𝝻l) per sample	2.4400	0.1075	0.0559	1.0850	3.2450	0.0064	−0.0001
Pricing	€€€(€)	€€€	€€	€	€€	€€€€	€€€€
Time efficiency	++	++	+	+++	+	++	+++
Diversity	+	+/−	++	–	+	NA	NA

€, most economic; €€€€, most exp ensive; NA, not applicable. € (euro) Specrum from € (least expensive) to €€€€ (most expensive) Spectrum of time efficiency: + time efficient protocol to +++ time consuming protocol Spectrum Diversity: - least diverse to ++ very diverse.

### Qualitative comparison

**Figure 3:**
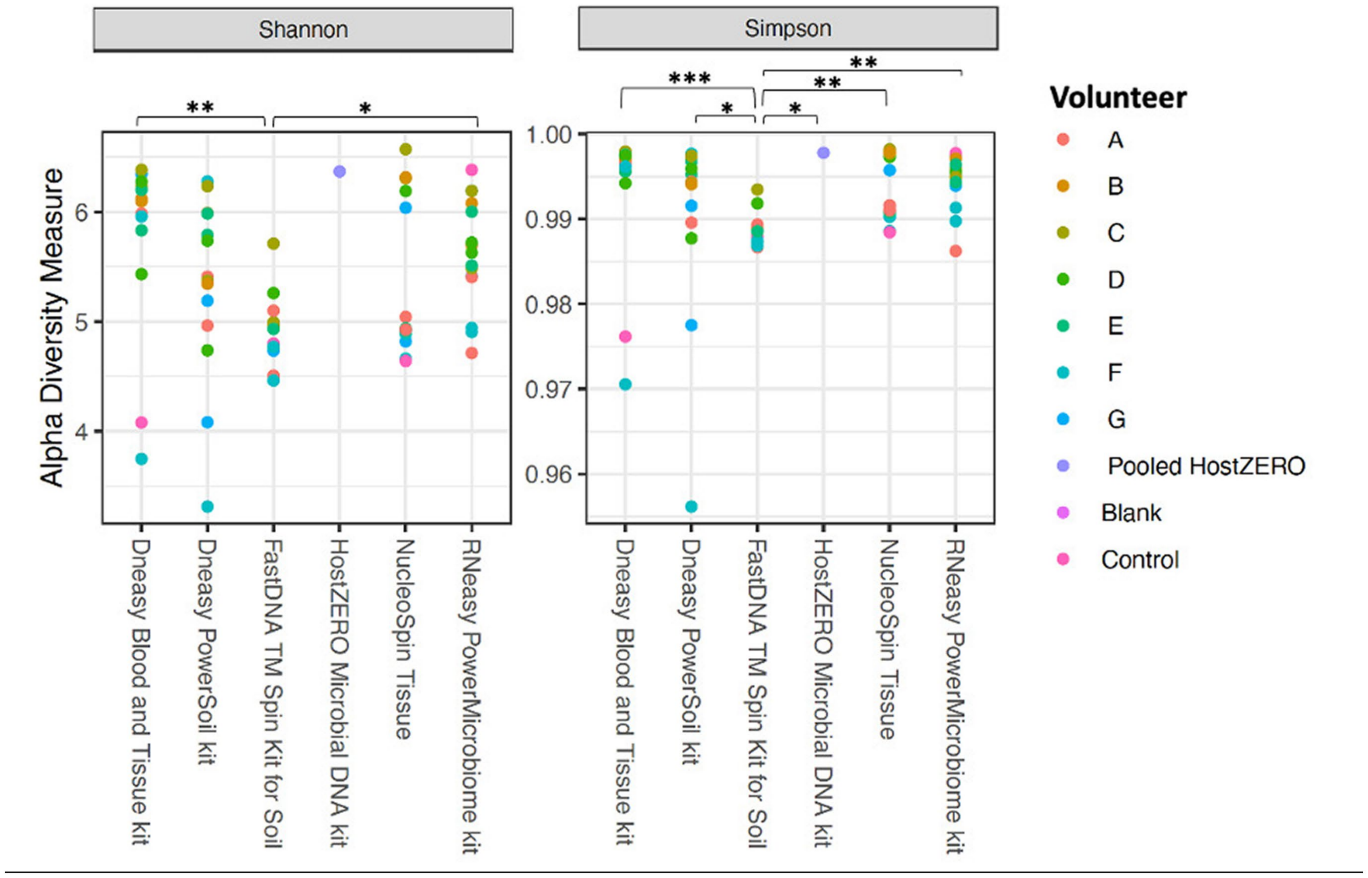
Alpha diversity measures (Shannon and Simpson diversity indices) per used extraction protocol. Shannon diversity was significantly lower for FastDNA compared to Blood & Tissue and RNeasy. When looking at Simpson diversity, FastDNA was significantly lower compared to Blood & Tissue, PowerSoil, HostZERO, NucleoSpin and RNeasy. There were no significant differences for observed richness. *p < 0.05, **p < 0.01, ***p < 0.001.

The alpha diversity measures Shannon and Simpson diversity were significantly different between the different extraction protocols (*p* = 0.003; *p* = 0.0002). Shannon diversity was significantly lower for FastDNA compared to Blood & Tissue and RNeasy; and when looking at Simpson diversity, FastDNA was significantly lower compared to Blood & Tissue, PowerSoil, HostZERO, NucleoSpin and RNeasy (Figure 3). There were no significant differences for observed richness.

Interestingly, samples analysed with the NucleoSpin protocol showed a gap in Simpson and Shannon diversity (Figure 3), with a separation between 10 samples having a lower Shannon and Simpson diversity and 6 samples displaying higher diversity. The protocol Blood & Tissue had the highest number of observed species, Shannon, and Simpson diversity, followed by RNeasy and PowerSoil (Table 4).

**Table 4:** Median or mean observed species, Shannon and Simpson diversity per extraction protocol.

		PowerSoil	NucleoSpin	Blood & Tissue	FastDNA	RNeasy
Observed	Median (IQR)		252 (236–759)	650 (583.0–703.5)		
	Mean (±SD)	387 (±238)			476 (±189)	456 (±231)
Shannon	Median (IQR)		4.9 (4.910–6.257)	6.1 (5.917–6.265)		
	Mean (±SD)	5.4 (±0.84)			4.9 (±0.30)	5.7 (±0.50)
Simpson	Median (IQR)	0.995 (0.990–0.997)	0.991 (0.991–0.998)	0.997 (0.996–0.998)	0.989 (0.988–0.989)	0.996 (0.994–0.997)

IQR, interquartile range; SD, standard deviation.

Of note, the right eye of volunteer F (no contact lens wearer, sleeps at her left side, rubs her eyes); Shannon, Simpson and observed richness were consistently within the lowest quartile with all used extraction protocols.

**Figure 4:**
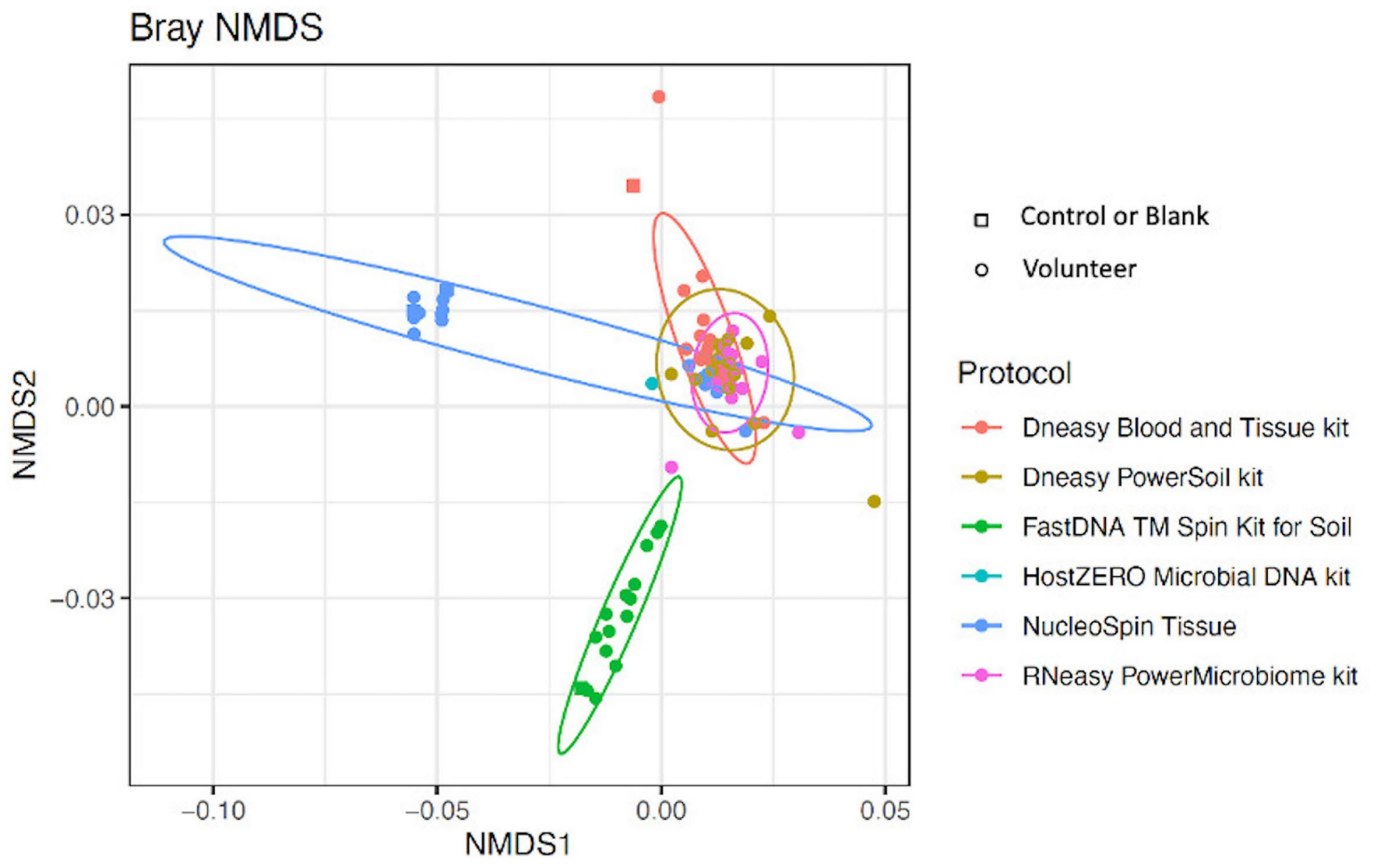
Bray-Curtis plot comparing different extraction protocols. Ordination diagram of nonmetric multidimensional scaling (NMDS), calculated based on the Bray–Curtis dissimilarity index showing clear microbial compositional differences dependent on the used extraction protocol.

Looking at the beta-diversity, visualized by the Bray-Curtis plot (Figure 4), clear microbial compositional differences - dependent on the extraction protocol used - were observed. RNeasy and PowerSoil are very alike with more variation in the PowerSoil analysed samples. Samples extracted with FastDNA displayed a different microbial profile compared to the samples analysed with the other extraction protocols. The samples analysed with NucleoSpin were separated based on their microbial composition, which was in line with what was observed for alpha diversity. The sample points located in the periphery are characterized by a high relative abundance of *Bacillus* (72% (0.7–0.73)) and *Flavobacterium* (25% (0.25–0.27)) whereas the centralised samples having a low abundance of *Bacillus* (0.87% ± 0.082) and *Flavobacterium* (0.28% ± 0.024) and a higher abundance of the core bacteria.

### Abundance levels per extraction protocol

Samples analysed with FastDNA have a clear predominance (i.e., present in all samples) for *Ralstonia* (relative abundance of 79%) and *Burkholderia-Caballeronia-Paraburkholderia* (4%) (both part of the family Burkholderiaceae). This explains the difference in microbial composition compared to other protocols. The pooled HostZERO samples have a relative abundance of ≥1% for most core bacteria; and a very high relative abundance of *Burkholderia-Caballeronia-Paraburkholderia*. No conclusions can be drawn on predominance as all samples were pooled. The genus *Bacillus* [72% (0.70 – 0.73)] and *Flavobacterium* (25% (0.25–0.27)) were highly abundant in 63% of samples analysed with the NucleoSpin protocol, the other 37% of samples had a microbial composition more similar to the samples analysed with Blood & Tissue (*Acinetobacter*: 3.2% (0.01–0.047), *Cutibacterium*: 21% ±0.08, *Corynebacterium*: 5.7% (0.049–0.063), *Pseudomonas*: 1% ± 0.006 *Staphylococcus*: 14% ± 0.13, *Streptococcus*: 10% (0.08–0.14). Not only the NucleoSpin protocol but also samples extracted with Blood & Tissue (8%) displayed a high abundance of *Bacillus* (Table 5).

**Table 5:** Comparison of relative abundances (≥1%) of the core genera as described in our systematic review [combining different extraction protocols (*n* = 359)] (Delbeke et al., 2021) (1), the healthy controls (*n* = 20) from our previous publication analysed with RNeasy (Delbeke et al., 2022) (2) and the sequencing results of our current project per used extraction protocol with and without decontamination steps.

			Not taking into account results of decontam						With potential contaminants excluded						QIAamp
Core bacteria	Core microbiome (*n* = 359) (1)	Rneasy (*n* = 20) (2)	Rneasy	FastDNA	B&T	NucleoSpin	PowerSoil	HostZERO	Rneasy	FastDNA	B&T	NucleoSpin	PowerSoil	HostZERO
*Acinetobacter*	6%	0.2%	0.8%	0.1%	4.7%	1.4%	1.8%	0.6%	2.24%	0.1%	6.92%	4.3%	NA	NA	NA
*Corynebacterium_1*	10%	29.0%	12.1%	1.1%	3.9%	2.3%	11.7%	1.8%	12.12%	1.23%	5.85%	7.5%	NA	NA	NA
Cutibacterium*	7%†	17.0%	32.2%	1.6%	15.7%	6.6%	24.3%	7.6%	12.84%	1.80%	3.88%	21.1%	NA	NA	NA
*Pseudomonas*	19%	0.1%	0.6%	3.1%	0.4%	0.2%	0.3%	2.9%	1.33%	0.35%	0.55%	0.5%	NA	NA	NA
*Staphylococcus*	6%	19.0%	17.9%	0.6%	11.0%	4.5%	9.0%	10.2%	12.95%	0.62%	15.8%	14.3%	NA	NA	NA
*Streptococcus*	3%	3.0%	8.5%	0.6%	6.0%	4.9%	12.5%	1.2%	2.19%	0.69%	7.4%	15.9%	NA	NA	NA
*Bacillus +*	8%	0.0%	0.1%	0.0%	7.6%	44.9%	0.2%	0.4%	0.13%	0.0%	2.44%	1.1%	NA	NA	NA
Bacteria with a relative abundance ≥ 1%		Rneasy (*n* = 20)	Rneasy	FastDNA	B&T	NucleoSpin	PowerSoil	HostZERO	Rneasy	FastDNA	B&T	NucleoSpin	PowerSoil	HostZERO	QIAamp
*Anaerococcus*		3.3%	1.4%	0.1%	0.5%	0.4%	1.7%	0.0%	2.78%	0.1%	0.73%	1.28%	NA	NA	NA
*Aquabacterium*		0.0%	0.1%	0.0%	1.0%	0.0%	0.0%	5.5%	0.24%	0.0%	1.51%	0.0%	NA	NA	NA
*Asaia*		0.0%	0.0%	0.0%	0.1%	0.0%	0.2%	1.1%	0.0%	0.0%	0.14%	0.0%	NA	NA	NA
*Burkholderia-Caballeronia-Paraburkholderia*		0.0%	0.3%	3.8%	1.0%	0.0%	0.1%	21.0%	0.80%	0.7%	1.55%	0.0%	NA	NA	NA
*Cupriavidus*		0.0%	0.0%	0.1%	0.0%	0.0%	0.0%	2.0%	0.0%	0.1%	0.0%	0.11%	NA	NA	NA
*Enhydrobacter*		0.5%	1.2%	0.1%	0.3%	0.3%	1.5%	0.5%	1.98%	0.12%	0.48%	0.74%	NA	NA	NA
*Erythrobacter*		0.0%	0.5%	0.0%	1.0%	0.0%	0.0%	0.0%	1.02%	0.0%	1.33%	0.1%	NA	NA	NA
*Ezakiella*		0.1%	0.5%	0.2%	0.2%	0.4%	2.1%	0.2%	1.59%	0.22%	0.31%	1.17%	NA	NA	NA
*Finegoldia*		1.5%	1.2%	0.1%	0.4%	0.4%	2.1%	0.2%	0.0%	0.15%	0.57%	1.00%	NA	NA	NA
*Flavobacterium*		0.0%	0.0%	0.0%	1.2%	22.7%	0.2%	0.5%	0.1%	0.0%	1.82%	0.14%	NA	NA	NA
*Herbaspirillum*		0.0%	0.0%	0.0%	0.0%	0.0%	0.0%	4.7%	0.0%	0.0%	0.0%	0.0%	NA	NA	NA
*Lawsonella*		1.9%	2.0%	0.5%	0.8%	1.0%	3.5%	0.1%	3.45%	0.6%	1.18%	3.09%	NA	NA	NA
*Lactobacillus*		0.0%	0.8%	0.2%	1.4%	0.2%	0.5%	4.1%	3.24%	0.2%	1.65%	0.61%	NA	NA	NA
*Janthinobacterium*		0.0%	0.0%	0.0%	0.0%	1.1%	0.0%	0.0%	0.0%	0.0%	0.0%	2.30%	NA	NA	NA
*Methylobacterium*		0.0%	0.0%	0.0%	0.0%	0.0%	0.1%	1.0%	0.0%	0.0%	0.1%	0.0%	NA	NA	NA
*Nitrospirillum*		0.0%	0.0%	0.0%	0.0%	0.0%	0.0%	5.7%	0.0%	0.0%	0.0%	0.0%	NA	NA	NA
*Pelagimonas*		0.0%	0.0%	0.0%	10.1%	0.0%	0.0%	0.0%	0.0%	0.0%	15.01%	0.0%	NA	NA	NA
*Peptoniphilus*		1.5%	0.8%	0.1%	0.3%	0.4%	1.6%	0.2%	1.94%	0.16%	0.50%	1.12%	NA	NA	NA
*Pelomonas*		0.0%	0.0%	0.2%	0.0%	0.0%	0.0%	1.9%	0.0%	0.17%	0.0%	0.0%	NA	NA	NA
*Rhodococcus*		0.0%	0.1%	0.0%	1.0%	0.1%	0.1%	0.3%	0.47%	0.0%	1.31%	0.2%	NA	NA	NA
*Ralstonia*		0.0%	4.0%	79.2%	0.0%	0.0%	0.3%	1.3%	7.33%	88.35%	0.0%	0.0%	NA	NA	NA
*Rothia*		0.3%	0.6%	0.1%	0.6%	0.3%	1.7%	0.3%	1.28%	0.1%	0.85%	1.01%	NA	NA	NA
*Sagittula*		0.0%	0.0%	0.0%	2.2%	0.0%	0.0%	0.0%	0.0%	0.0%	0.0%	0.0%	NA	NA	NA
*Sulfitobacter*		0.0%	0.0%	0.0%	2.6%	0.0%	0.1%	0.3%	0.0%	0.0%	3.34%	0.0%	NA	NA	NA

+*Bacillus* did not meet the criteria of the core microbiome in our review paper (Delbeke et al., 2021), but was quite abundant when present in the reviewed papers. ^*^In previously published ocular surface microbiome research, *Cutibacterium* was not mentioned. This can be explained by the reclassification of 10 species from the genus *Propionibacterium* to, among others, *Cutibacterium*. This explains why our review paper describes *Propionibacterium* (7%) instead of *Cutibacterium* (Delbeke et al., 2021). NA, not applicable. The genera with a relative abundance ≥1% were listed and the abundance of each genus retrieved with other extraction protocols was added as a comparator.

### Repeatability

**Figure 5:**
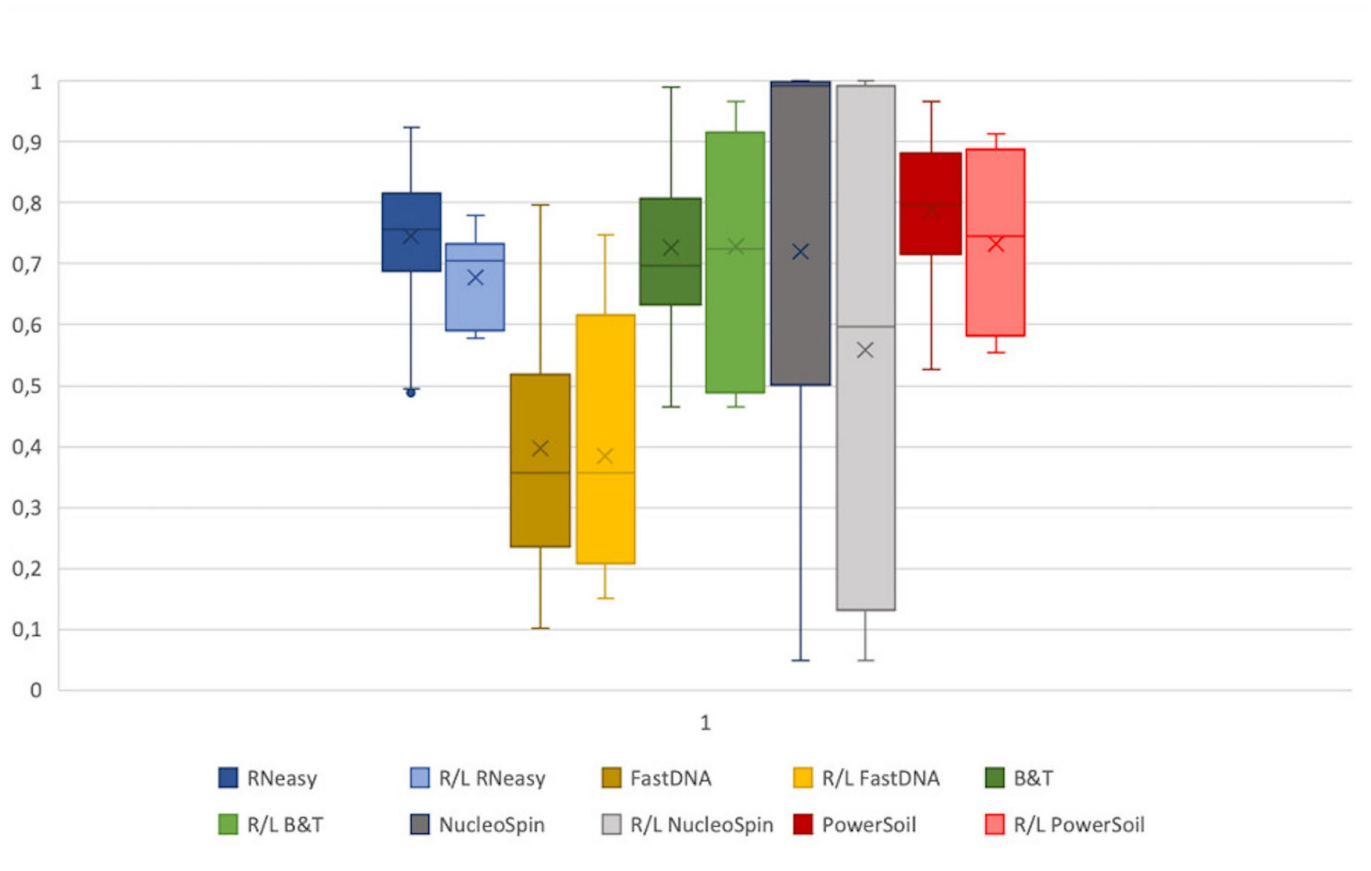
Extraction protocol repeatability. The Bray-Curtis distances of the samples analysed with a certain extraction protocol were visualized next to the distance of the right and left eye of every volunteer per protocol. The distance between all samples analysed with a certain protocol were not significantly different from the distance between the right and left eye. Based on the within subject distance, FastDNA is the most repeatable (0.39 ± 0.2), followed by NucleoSpin (0.56 ± 0.37) and RNeasy (0.68 ± 0.08).

Assuming, based on the publication of Cavuoto et al. (2018), a similar microbial composition of the right and left eye within a subject; a low within-subject distance, consistent for all sampled volunteers, would reflect high repeatability of a protocol (Figure 5). No significant difference between within-subject similarity and overall distance were observed for any of the protocols assessed. However, there was a non-significant trend of lower distance within a subject compared to the overall distance in the samples analysed with RNeasy (*p* = 0.06).

Based on the within-subject distance, FastDNA had the most repeatable results (0.39 ± 0.2), followed by NucleoSpin (0.56 ± 0.37) and RNeasy (0.68 ± 0.08). The high repeatability of the samples analysed with the FastDNA protocol can be explained by the predominance for the genus *Ralstonia*. NucleoSpin analysed samples are very repeatable in some volunteers, but in other; the right and left eye are very dissimilar (e.g., volunteer G and D). This (dis)similarity seems to be related to the presence of the genus *Bacillus*.

### Reference frame

**Figure 6:**
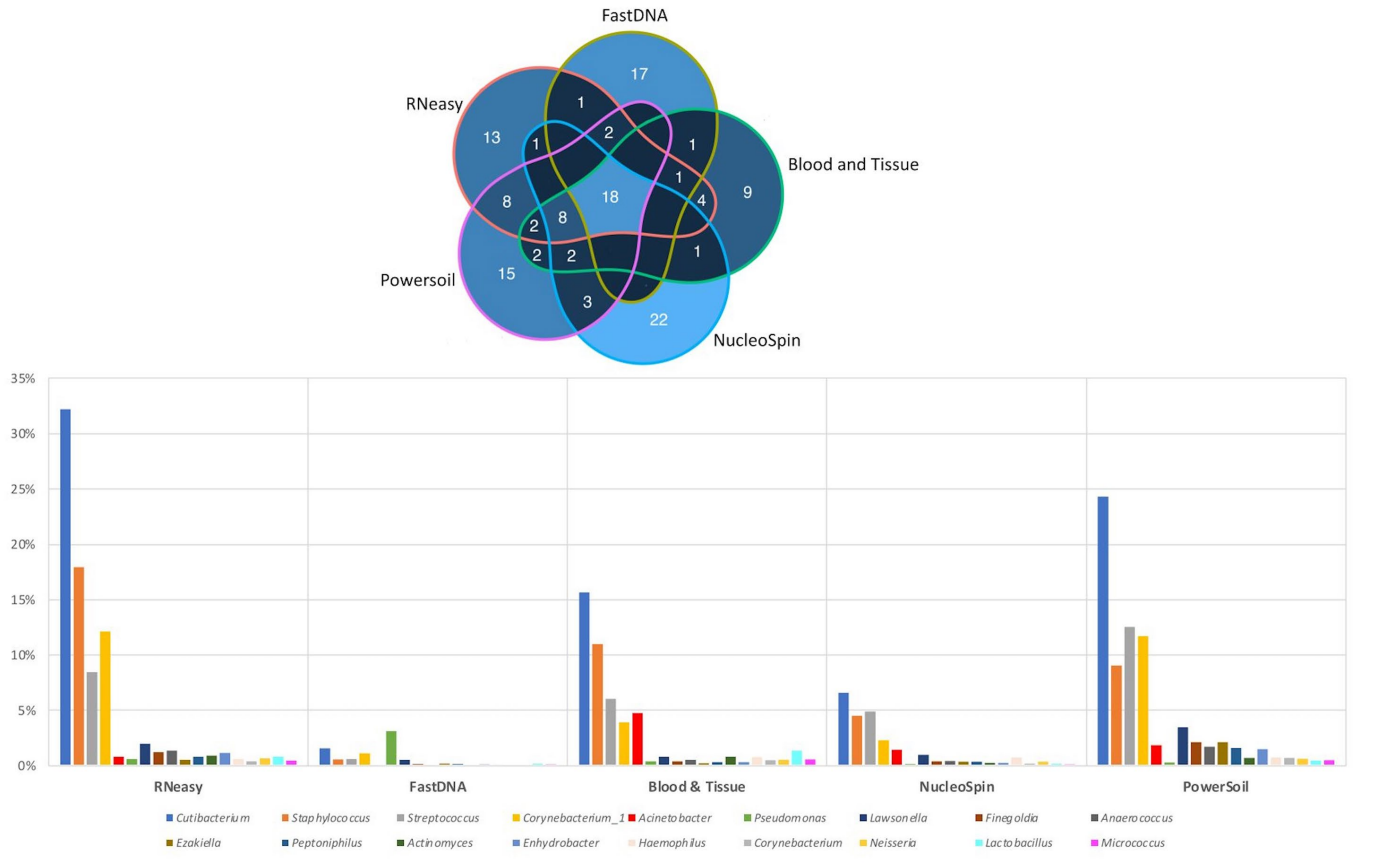
Top 20 genera per volunteer per extraction protocol. Top: Venn diagram depicting the number of overlapping genera between different extraction protocols (not taking into account the host DNA depletion kits). Calculated by looking at the top 20 per volunteer per protocol, 18 genera were extracted by all protocols. Bottom: Distribution, by means of relative abundance, of the 18 genera in common per extraction protocol. The six core genera as described in our earlier paper (Delbeke et al., 2021) can be extracted by all protocols. Bacillus was not part of these 18 genera.

The 20 most abundant genera per volunteer per extraction protocol were listed and compared (Figure 6). We did not include the HostZERO and QIAamp kits since those samples were pooled before sequencing, so statements on individual level were not possible. We found 18 genera in common, 6 of those were the core bacteria as described earlier (Delbeke et al., 2021; Figure 6). *Cutibacterium, Staphylococcus, Streptococcus* and *Corynebacterium_1* had the highest abundance of all 18 overlapping genera independent of the used extraction protocol (bottom Figure 6).

### Discriminative features

LEfSe analysis confirmed the difference in relative abundance of certain bacteria dependent on the extraction protocol used. *Bacillus* and *Flavobacterium* were discriminative genera for NucleoSpin, as was *Ralstonia* for FastDNA (Table 1). The discriminative genera of RNeasy and PowerSoil were bacteria from the core microbiome. The discriminative genera of Blood & Tissue were mainly *Sultifobacter* and *Pelagminoas*. Interestingly, LEfSe analysis showed no discriminative genera between RNeasy and PowerSoil, which is in line with the results of the Bray-Curtis plot (Figure 4).

Extraction protocols were also grouped based on type of lyses (mechanical or chemical) (Table 2) resulting in different discriminative genera (Supplementary 2).

### Potential contaminants

The control swab of the PowerSoil analysed samples failed to construct a library, making it impossible to apply the decontam package to this protocol. As both host DNA depletion kits were pooled, these samples were also not fit for further decontamination steps.

Different methods from the decontam R package were applied. All ASVs within the “prevalence” group with default threshold 0.1 were also present in the “prevalence” group with threshold 0.5. Furthermore, there were also some overlapping ASVs between the “frequency” and “prevalence” group. Supplementary 3 gives an overview of both the number of ASVs per used method, and the most important ASVs assigned as potential contaminant per extraction protocol.

Removing these contaminants had a clear influence on the relative abundances of the samples analysed with NucleoSpin protocol (Table 5). The removal of *Bacillus* ASVs (Table 5) consequently raised the relative abundance of the majority of core bacteria (*Cutibacterium*, *Corynebacterium_1*, *Acinetobacter*, *Staphylococcus* and *Streptococcus*). There was a lowering in relative abundance of *Burkholderia-Caballeronia-Paraburkholderia* and *Pseudomonas* in the FastDNA analysed samples; but *Ralstonia* remained very abundant, despite the elimination of 95 ASVs annotating for this genus.

The Bray-Curtis plot comparing different extraction protocols was recalculated after removing the potential contaminant ASVs (Supplementary 5), showing that the kit specific differences remained.

## Discussion

We performed a comparative study to build a reference frame to allow the comparison and interpretation of 16S rRNA amplicon sequencing studies on the ocular surface microbiome using different DNA extraction protocols.

As visualized in Figure 2 and Table 3; RNeasy and PowerSoil have the highest bacterial DNA yield. A very low DNA yield was retrieved with the two host DNA depletion kits, HOST ZERO and QIAamp. For host DNA depletion kits, it was recently put forward that freeze-thawing samples would compromise the integrity of the bacterial cell wall, exposing bacterial DNA along with the host DNA during the host enzymatic depletion step. This detrimental effect on bacterial DNA, would moreover be especially of importance in low biomasses, such as ocular samples (Shi et al., 2022; Wiscovitch-Russo et al., 2022). This is in line with our own observations, that bacterial DNA is extracted less effectively or destroyed alongside with the host DNA. Wiscovitch-Russo and his co-workers advise to use fresh samples with host DNA depletion kits to avoid this freeze-thawing process (Wiscovitch-Russo et al., 2022). Saladié et al. compared 3 extraction protocols on bronchoalveolar lavage fluid, another low biomass sample type, and likewise detected a significantly lower number of 16S copies when using the commercial QIAamp both with and without host DNA depletion, compared to a newer extraction method using polyethylene glycol (Saladié et al., 2020).

As not all bacteria within a complex microbial community can be extracted equally efficient with each DNA extraction protocol, the choice of the extraction protocol should be tailored to the research question at hand. Based on both abundance levels (Figure 6 and Table 5) and results of the LEfSe analyses (Table 1); *Bacillus* has a high abundance in the NucleoSpin (relative abundance 45%), and Blood & Tissue (relative abundance 8%) extracted samples. Extrapolating our observation with published literature confirmed a *Bacillus* tendency of different Macherey – Nagel NucleoSpin protocols (6%: Ozkan et al., 2019; 2%: Wang et al., 2021). Ozkan et al. (2019) and Wang et al. (2021) used the NucleoSpin Tissue XS kit and NucleoSpin 96 Soil DNA Isolation Kit, respectively. The NucleoSpin Tissue and the NucleoSpin Tissue XS kit have the same buffer chemistry and workflow; the only difference lays in the elution buffer [which we have lowered in our set-up to the same amount as used in the XS kit (Supplementary 1)] and the reduced surface of the membrane. This reduced membrane surface has the advantage of recovering lower yields with a higher rate. The NucleoSpin 96 Soil is made for the simultaneously work with up to 96 samples at a time and uses different lysis buffers.

RNeasy, PowerSoil and to a lesser extent Blood & Tissue seem to extract the core genera more easily (Figure 6; Table 5). The lack of discriminating features on LEfSe analysis between RNeasy and PowerSoil extracted samples substantiates their similarity.

*Sulfitobacter* and *Pelagimonas* were the discriminative genera for the Blood & Tissue extracted samples with, respectively, relative abundances of 10 and 2.6% (remaining 15 and 3%, respectively, after removing the 246 ASVs based on the decontam package). This extraction protocol was added to our comparison based on the publication of Doan et al. (2016). However, those two genera were not mentioned in their list of bacteria with a relative abundance ≥0.1%. *Sulfitobacter* and *Pelagimonas* are to our knowledge also not known as potential contaminants of low biomasses. Previously, both have been extracted from ocean water (Olesen et al., 2016) and have to our knowledge not been reported in human samples so far.

Sequencing studies on population level (i.e., how the ocular surface microbiome looks like in a certain population) asks for an extraction protocol that represents the samples as diverse as possible in a repeatable and cost and time efficient way (Table 3). Firstly, Blood & Tissue, PowerSoil and RNeasy extracted samples have the most diverse bacterial population. Furthermore, the overall relative abundances of the samples analysed with Blood & Tissue and RNeasy were only minimally impacted by additional decontamination steps (Table 5). When looking at repeatability next (Figure 5), FastDNA clearly stands out. Albeit this is merely due to the marked predominance for *Ralstonia* and *Burkholderia-Caballeronia-Paraburkholderia*, making samples more alike. NucleoSpin is very repeatable in some samples, but in other volunteers, the right and left eye are very dissimilar and this large difference in repeatability can be reduced to the presence or not of *Bacillus*. RNeasy scores consistently good with a low standard deviation and low interquartile range, meaning there is high consistency between the different volunteers. Of note, the relative abundances of the samples extracted with RNeasy are very similar to our previously described results where volunteers were sampled under general anaesthesia (Delbeke et al., 2022; Table 5). This underscores the repeatability of this extraction protocol and also highlights that the difference in sampling depth (soft versus firm swabbing), as earlier described, might only be a minor contributor to the retrieved sequencing differences in different publications (Dong et al., 2011).

Finally, with respect to costs, the host DNA depletion kits are overall the most expensive and more time consuming. The NucleoSpin protocol is well-priced but was more time-consuming which resulted in a higher manpower cost.

As mentioned above and as shown in Figure 4, there is a clear compositional difference in the samples analysed with the NucleoSpin protocol. The sample points located in the periphery are characterized by a high relative abundance of *Bacillus* (72% (0.7–0.73)) and *Flavobacterium* (25% (0.25–0.27)) whereas the centralised samples having a low abundance of *Bacillus* (0.87% ± 0.082) and *Flavobacterium* (0.28% ± 0.024) and a higher abundance of the core bacteria. This also explains the gap in both Shannon and Simpson diversity (Figure 3) and the large difference in repeatability (Figure 5). These results align with earlier research in stool samples (NucleoSpin® DNA stool kit), showing that the results of samples analysed with the NucleoSpin stool kit were inconsistent and dependent on the DNA yield. According to the authors’, it is more difficult to successfully extract the bacterial DNA with the NucleoSpin stool kit in samples with a low bacterial load (as is the case in eye samples) (Fiedorová et al., 2019). However, next to extraction efficiency; this large variability might also be due to contaminants. A large drop was encountered in the relative abundance of *Bacillus* (45 to 1%) and *Flavobacterium* (23%–0.1%) in the NucleoSpin analysed samples after removing all ASVs retrieved with decontam (*n* = 246). This hypothesis is further underscored by a previous publication showing that *Flavobacterium* and to a much lower extent *Bacillus*, are typical genera present in reagents (Weyrich et al., 2019). This lines up with our blank NucleoSpin swab showing a high number of reads for both *Flavobacterium* and *Bacillus*. Moreover, a publication on children’s stool samples already showed the ability of RNeasy – one of our other protocols used - to efficiently extract *Flavobacterium* (Peterson et al., 2021). This last argument implies that the abundance difference is not merely due to the other extraction kits not being able to extract this particular genus; but that *Flavobacterium* might be a contaminant.

Overall, when looking at the Bray-Curtis plots with and without potential contaminants, the extraction kit specific compositional differences remained present and the effect of the extraction kit on the sequencing results transcends the inter-individual differences (Figure 4; Supplementary 5).

When looking at the 20 most abundant genera per volunteer, per extraction protocol; 18 genera were extracted by all protocols (not taking-into-account the human depletion kits as those were pooled) (Figure 6). Those 18 genera were *Cutibacterium, Staphylococcus, Streptococcus, Corynebacterium_1, Acinetobacter, Pseudomonas, Lawsonella, Finegoldia, Anaerococcus, Ezakiella, Peptoniphilus, Actinomyces, Enhydrobacter, Haemophilus, Corynebacterium, Neisseria, Lactobacillus, Micrococcus*; with the first 6 being the core bacteria (Delbeke et al., 2021). *Bacillus* could not be extracted with all DNA extraction protocols, which is in line with our earlier publication (Delbeke et al., 2021), stating that *Bacillus* was present with a high abundance in only certain publications (Zhou et al., 2014; Ozkan et al., 2017; Li Z. et al., 2019; Li S. et al., 2019; Ozkan et al., 2019; Wang et al., 2021). We extracted *Bacillus* with NucleoSpin, Blood & Tissue and to a far lesser extent with PowerSoil; which is consistent with Ozkan et al. and Wang et al. retrieving *Bacillus* using different Macherey – Nagel NucleoSpin protocols (Ozkan et al., 2017, 2019; Wang et al., 2021) and Zhou et al. (2014) using the PowerSoil extraction protocol. Interestingly and as briefly stated above, there is a large drop in relative abundance of *Bacillus* in the NucleoSpin (45–1%) and Blood & Tissue (8–2%) analysed samples after removing all ASVs retrieved with decontam (NucleoSpin *n* = 246, Blood & Tissue *n* = 203). Similarly, Ozkan and his co-workers (NucleoSpin Tissue XS kit) used indicspecies R package and removed all operational taxonomic units (counterpart of ASV) present in their negative controls. Despite this contamination step, they still retrieved *Bacillus* (Ozkan et al., 2019). The FastDNA analysed samples, in contrast, had a relative abundance of *Bacillus* below <0.1%, which is in line with a publication of Mc Orist et al. showing that NucleoSpin seem to more easily extract gram-positive bacteria – as is *Bacillus* - compared to FastDNA (of note, they analysed a pure bacterial culture of the Gram-positive *Lactobacillus acidophilus*) (McOrist et al., 2002). Future research needs to clarify if this is a true contaminant or if it is of real importance, as this genus also had a low relative abundance in samples analysed with other extraction kits. An alternative explanation has been described by Borroni et al. with his theory of an eye community state type (ECST) (Borroni et al., 2022). Each ECST is defined by a different set of microorganisms. One particular ECST (type 7) seems to have a high abundance of *Bacillus*. This theory elucidates the absence of *Bacillus* in the paper of Andersson and her colleagues, who also used the NucleoSpin Tissue XS kit (Andersson et al., 2021).

Borroni et al. used in their publication the QIAamp DNA Microbiome Kit. We have used this protocol with the host DNA depletion steps. These additional host DNA depletion steps lowered the amount of DNA leading to the pooling of the different samples. This pooled sample failed to construct a library, making sequencing impossible. As nothing was mentioned in the Borroni paper on host DNA depletion, the assumption can be made that they have extracted their samples with the QIAamp DNA Microbiome Kit without host DNA depletion (Borroni et al., 2022).

Critical interpretation of sequencing results and taking into consideration the presence of contaminants especially in a low biomass such as the eye surface is of utmost importance. The abundance of *Ralstonia* was very high in both the conjunctival as control samples analysed with FastDNA, but the abundance of Ralstonia remained high, even after removing the ASVs attributed as potential contaminants. However, *Ralstonia* was the main laboratory contaminant in a lower respiratory tract study (also being a low bacterial load sample) (Drengenes et al., 2019) and Salter et al. also highlighted *Ralstonia* as a typical contaminant in their blank controls (of which most samples were extracted with FastDNA) (Salter et al., 2014). *Ralstonia* was also the major taxon in the negative controls of Zhou et al., using the PowerSoil DNA isolation kit (Zhou et al., 2014). In the publication of Ham et al. (i-genomic Soil DNA Extraction Mini Kit), *Ralstonia* was present with a relative abundance of 4% (negative controls were not sequenced based on the very low DNA yield) (Ham et al., 2018). Retuerto and his co-workers found an abundance of *Ralstonia* > 70% across their samples (i.c. contact lenses) using the QIAamp Fast DNA extraction kit (Qiagen, Hilden, Germany). We also would like to highlight that *Ralstonia* used to be categorised under the genus *Pseudomonas* (Zhou et al., 2001; Retuerto et al., 2019), this might have led in older publications to an overrepresentation of the abundance of *Pseudomonas* in disadvantage of *Ralstonia*.

*Ralstonia* was not only present in the FastDNA extracted samples. As visualized in Table 5, the RNeasy extracted samples showed a relative abundance of *Ralstonia* of 4% (7% after decontamination) and this can exclusively be attributed to one sample (the left eye of volunteer E). The presence of *Ralstonia* in the left eye of this volunteer could not be confirmed with the other extraction protocols (with the exception of FastDNA where all samples had a high abundance of *Ralstonia*).

Wang et al. (2021) (NucleoSpin) found that *Ralstonia* might be a biomarker for mixed blepharitis. Further research is needed to elucidate a day-by-day variation in *Ralstonia* abundance (explaining why 1 volunteer had a high abundance in only 1 swab) and if there is any correlation with a variable presence of blepharitis; or if it is merely a contaminant.

Confounders were considered by using different extraction protocols on samples taken on the same day of different volunteers (Figure 1). This strategy was chosen in order to dilute potential influence of environmental and/or weather conditions on the microbial composition. Repeated sampling was not performed on consecutive days to reduce possible confounding, such as ocular surface irritation, due to repetitive sampling. All volunteers were within a narrow age range (22–25 years old) to avoid the introduction of sequencing differences due to different age groups. Furthermore, although there seems to be a high level of concordance in microbiome composition in the same-person eyes (Delbeke et al., 2022) and the immunological and genetic background in one person are the same; the environmental factors (such as rubbing or sleeping position) may differ between eyes. To account for those potential confounders, all volunteers were asked to fill in a small questionnaire. Unfortunately, due to the limited number of included volunteers and the large variability in answers, it is impossible to draw any conclusions. Larger studies are needed to further clarify the importance of confounding factors. Although our set-up corrected multiple confounding factors, the temporal variation of the ocular surface microbiome could not be accounted for (Ozkan et al., 2017). In faeces samples, one sample of one volunteer can be used to test different extraction protocols; the low biomass of the ocular surface needs more inventive measures. Overall, the repeatability of our results per extraction protocol seems to transcend the potential effect of the day-by-day variation.

We have used ocular surface samples to compare the different extraction protocols. An alternative approach can be seen in the use of a controlled mock microbial community with known levels of gram-positive and gram-negative bacteria. The rationale for not using a mock microbial community lies in the insufficient insights in the true microbial composition of the ocular surface. The goal of this research project was to compare different extraction protocols on real live low biomass samples of the ocular surface; as extraction efficiency differs depending on the used sample (faeces versus oral versus ocular). A mock microbial community could have an added value as it would provide additional data on DNA extraction, environmental and DNA reagents.

Our research is limited by the sparse amount of bacterial DNA on the ocular surface. This forced us to use inventive measures to test repeatability. We compared the right and the left eye of the same volunteer as an approximation, based on a study of Cavuoto et al. (2018), but larger population studies are needed to substantiate this. Low biomasses make 16S rRNA sequencing challenging. The host DNA depletion kits yielded a very low amount of DNA and the samples extracted with QIAamp even failed to construct a library. Latter can be explained by either inhibition due to certain substances or by the very low yield of bacterial DNA. Moreover, we only looked at commercially available extraction kits as they are more easily reproducible. However, many publications use lab-made extraction kits (Lee et al., 2012; Aoki et al., 2013; Zhou et al., 2014). One of the standard extraction procedures used in microbiome studies is the phenol chloroform isoamyl alcohol method. Previous research has shown that this standard technique is not ideal for low biomass samples due to the multiple treatment and washing steps and the risk of phenol being carried over potentially interfering the downstream processing (Wiscovitch-Russo et al., 2022).

Furthermore, little is known on the importance of contaminants in 16S rRNA research of the ocular surface. Our project is to our knowledge the first that looks deeper into the role of contaminants in ocular surface next generation sequencing research, based on differently used extraction protocols. Validation of our results is needed to truly know the impact of genera such as *Ralstonia*, *Burkholderia-Caballeronia-Paraburkholderia*, *Bacillus*, *Flavobacterium*, *Sultifobacter* and *Pelagimonas* among others.

Lastly, it is worth mentioning that sequencing methods are multiple biases potential biases introduced during the whole process. These biases can range from different collection techniques [e.g., swab (Delbeke et al., 2022) versus tears (Willis et al., 2020) versus contact lens (Retuerto et al., 2019) or tissue (Ozkan et al., 2018)], to the use of different storage types and storage duration. The effect of the DNA extraction protocol has been discussed in detail in this paper, but also the choice of which hypervariable region of the 16S rRNA gene is targeted influences the sequencing results (Boers et al., 2019). Further in the process, sequencing errors or differently used denoising (e.g., MOTHUR or USEARCH (Delbeke et al., 2021)) and/or clustering algorithms should be taken into account. Until recently, it was common practice to cluster the obtained sequence reads into operational taxonomic units (OTUs). These OTUs are clusters of sequences at a certain sequence similarity threshold. The most commonly used similarity threshold is 97%; in other words, a distance of 3% (Westcott and Schloss, 2015). However, OTUs miss real biological sequence variation which has led to the development of amplicon sequence variants (ASVs). ASVs distinguish sequence variants differing by as little as one nucleotide, leading to a higher resolution (Callahan et al., 2017). Albeit this higher resolution should also be used with caution, as they still do not represent a meaningful taxonomic unit (Schloss, 2021). Furthermore, depth bias; a less obvious confounder, can prevent the revealing of minority populations that are present at concentrations lower than 10^5^/mL (Lagier et al., 2012). Lastly, the chosen taxonomic classifier (e.g., Greengenes, SILVA or RDP) and bio-informatic pipeline both impact sequencing result.

In conclusion, many high-quality papers have been published in the field of the ocular surface microbiome, but consensus on the importance of the used extraction protocol is lacking. Our prospective comparative study builds a reference frame to facilitate the interpretation of currently published papers and to ease the choice of the extraction protocol in the future based on the research question. To our knowledge, this project is the first to explore deeper the role of contaminants in ocular surface microbiome research.

## Data availability statement

The datasets presented in this study can be found in online repositories. The names of the repository/repositories and accession number(s) can be found at: https://dataview.ncbi.nlm.nih.gov/object/PRJNA912212?reviewer=rp89vnp3d6shd37ruh9vffdp1n, PRJNA912212.

## Ethics statement

The studies involving human participants were reviewed and approved by Ethics Committee Research UZ/KU Leuven, Belgium. The patients/participants provided their written informed consent to participate in this study.

## Author contributions

All authors listed have made a substantial, direct, and intellectual contribution to the work and approved it for publication.

## Funding

This paper was funded by the University Hospitals Leuven, Belgium *via* the “funding for academic research” (“fonds voor academisch onderzoek”).

## Conflict of interest

The authors declare that the research was conducted in the absence of any commercial or financial relationships that could be construed as a potential conflict of interest.

## Publisher’s note

All claims expressed in this article are solely those of the authors and do not necessarily represent those of their affiliated organizations, or those of the publisher, the editors and the reviewers. Any product that may be evaluated in this article, or claim that may be made by its manufacturer, is not guaranteed or endorsed by the publisher.

## Supplementary material

SUPPLEMENTARY 1 DATA SHEET 3 Protocol adjustments added at our own discretion or chosen based on themanufacturer’s guidelines.

SUPPLEMENTARY 2 FIGURE 2 LefSe analysis comparing chemical and mechanical lysis.

SUPPLEMENTARY 3 DATA SHEET 2 The number of ASV per used method applied in the decontam package in R, and the most important ASV that were assigned as potential contaminant per extraction protocol.

SUPPLEMENTARY 4 DATA SHEET 2 DNA concentration per protocol with and without negative controls and blank, before and after concentration (ng/μL).

SUPPLEMENTARY 5 FIGURE 1 Bray-Curtis plots comparing different extraction protocols after removing the potential contaminant ASVs.Ordination diagram of nonmetric multidimensional scaling (NMDS), calculated based on the Bray-Curtis dissimilarity index after removing the potential contaminants. Interestingly, the extraction kit specific compositional differences remained present after decontamination. It needs to be taken into account that no decontamination was applied.
