# Supplementary material for: DNA extraction protocol impacts ocular surface microbiome profile
Source: Front Microbiol. 2023 Apr 20;14:1128917. doi: 10.3389/fmicb.2023.1128917 (PMC10157640; doi:10.3389/fmicb.2023.1128917)
Supplement: SUPPLEMENTARY 1 DATA SHEET 3 — Protocol adjustments added at our own discretion or chosen based on themanufacturer’s guidelines. [file Data_Sheet_3.PDF]

Protocol	Adjustments added or chosen based on the manufacturer's guidelines
PowerSoil	30 µl of C1 was added to the Powerbead tubes, brush was carefully transferred without touching
	30 µl of C1 was added to the Eppendorf tube where the swab was preserved, subsequently the tube was vortexed, and solution was transferred to the Powerbead tubes
	Solution C6: 40 µl in stead of 100 µl
Blood & Tissue (protocol for animal blood or cells; cultured cells)	150 µl NaCl was added to Eppendorf tubes containing the sample, subsequently the tube was vortexed followed by short spin en solution was transferred to fresh tubes - 5000 xg for 10 minutes - removal of supernatans
	Two elutions
NucleoSpin	Buffer BE: 30 µl in stead of 100 µl; 5 min of incubtion at room temperature in stead of 1 min
	Incubated at 56° for 2 hours
RNeasy (3)	DNase and β-mercapto-ethanol steps were omitted
	325 µl PM1 was added to the PowerBead tube, brush was carefully transferred without touching
	325 µl PM1 was added to the Eppendorf tube were the swab was preserved, subsequently the tube was vortexed, and solution was transferred to the Powerbead tubes
	After vortexing the PowerBead Tubes for 10 min, the tubes were placed at 90° for 10 min to add a heat lysis step
	Final DNA was dissolved in 50 µl DNase-Free Water instead of the standard 100 µl
	Before final centrifugation, the collection tubes with filter membrane were incubated at room temperature for 5 min instead of 1 min
HostZERO	ZymoBIOMICS® DNase/RNase Free Water: 30 µl in stead of 20 µl
FastDNA	578 µl Sodium Phosphate Buffer was added to Lysing Matrix E tube, brush was carefully transferred without touching
	400 µl Sodium Phosphate Buffer was added to the Eppendorf tube where the swab was preserved, subsequently the tube was vortexed, and solution was transferred to the Lysing Matrix E tube
QIAamp	250 µl NaCl was added to a new Eppendorf tube, brush was carefully transferred without touching and both were vortexed
	250 µl NaCl was added to the Eppendorf tube where the swab was preserved, subsequently the tube was vortexed, and solution was transferred to the new Eppendorf tube
