# Supplementary figures and images for: DNA extraction protocol impacts ocular surface microbiome profile
Source: Front Microbiol. 2023 Apr 20;14:1128917. doi: 10.3389/fmicb.2023.1128917 (PMC10157640; doi:10.3389/fmicb.2023.1128917)

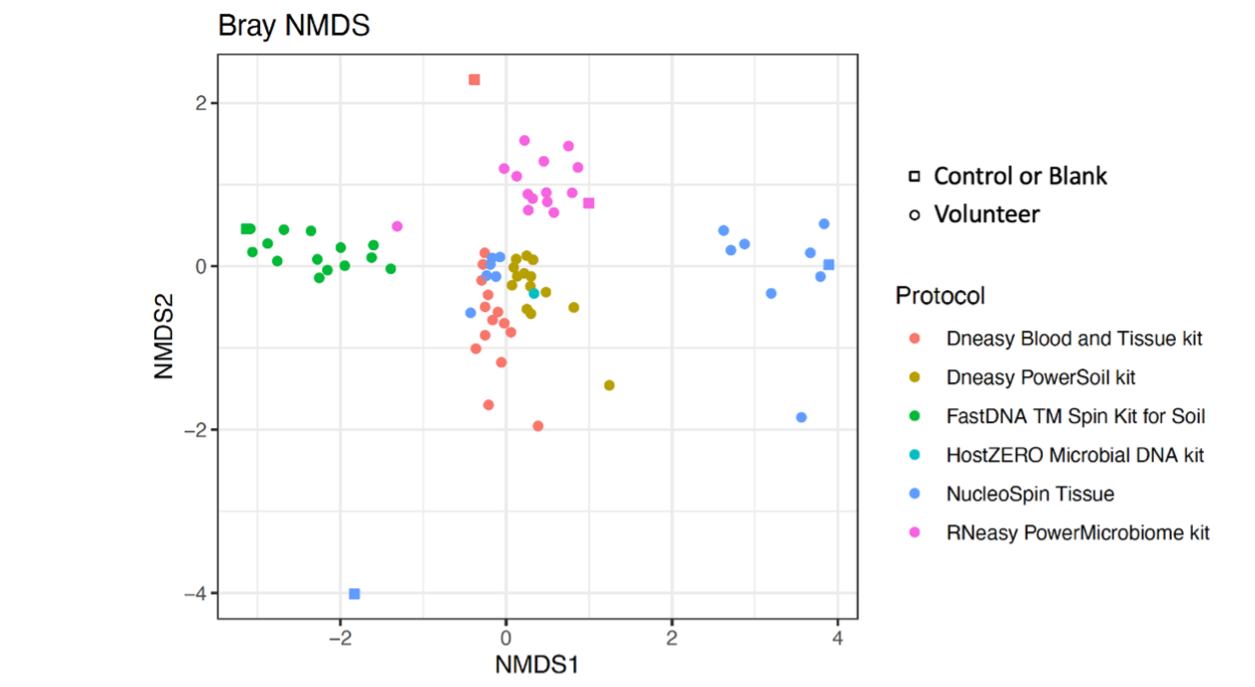

Supplement: SUPPLEMENTARY 2 FIGURE 2 — LefSe analysis comparing chemical and mechanical lysis. [file Image_1.PNG]

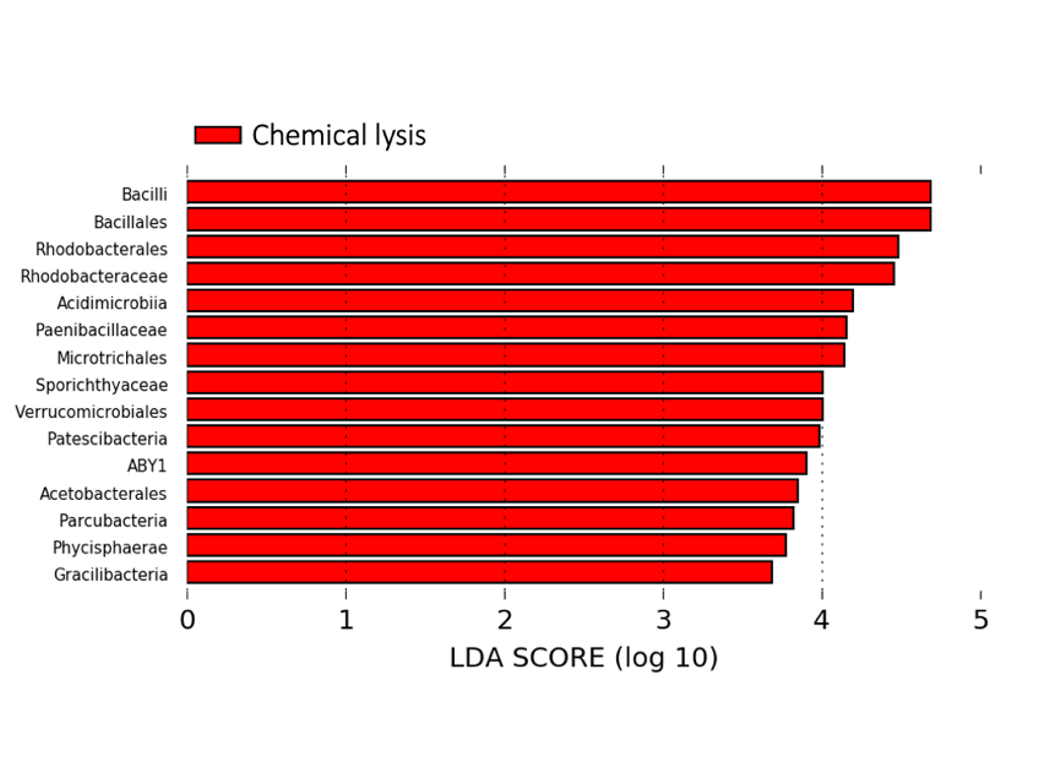

Supplement: SUPPLEMENTARY 5 FIGURE 1 — Bray-Curtis plots comparing different extraction protocols after removing the potential contaminant ASVs.Ordination diagram of nonmetric multidimensional scaling (NMDS), calculated based on the Bray-Curtis dissimilarity index after removing the potential contaminants. Interestingly, the extraction kit specific compositional differences remained present after decontamination. It needs to be taken into account that no decontamination was applied. [file Image_2.PNG]
