# Supplementary material for: DNA extraction protocol impacts ocular surface microbiome profile
Source: Front Microbiol. 2023 Apr 20;14:1128917. doi: 10.3389/fmicb.2023.1128917 (PMC10157640; doi:10.3389/fmicb.2023.1128917)
Supplement: SUPPLEMENTARY 3 DATA SHEET 2 — The number of ASV per used method applied in the decontam package in R, and the most important ASV that were assigned as potential contaminant per extraction protocol. [file Data_Sheet_2.PDF]

| | Method used in R | | | | Combining all methods | |
|---|---|---|---|---|---|---|
| | Frequency (n ASV) | Prevalence (n ASV) | Prevalence with threshold 0.5 (n ASV) | Both (n ASV) | n ASV | Most prominent potential contaminants per extraction protocol |
| RNeasy | 29 | 191 | 441 | 0 | 457 | *Streptococcus* (n=89), *Cutibacterium* (n=78), *Staphylococcus* (n=51),*Corynebacterium_1* (n=41), *Brevundimonas* (n=32),*Burkholderia-Caballeronia-Paraburkholderia* (n=32), *Finegoldia* (n=29), *Achromobacter* (n=20), *Curvibacter* (n=20), *Haemophilus* (n=25) |
| FastDNA | 10 | 10 | 337 | 0 | 347 | *Burkholderia-Caballeronia-Paraburkholderia* (n=162), *Ralstonia* (n=95), *Pseudomonas* (=35 ASV), *Reyranella* (n=24), *Lactobacillus* (n=16) |
| Blood & Tissue | 35 | 3 | 168 | 0 | 203 | *Cutibacterium* (n=74), *Bacillus* (n=36), *Sagittula* (n=34), *Streptococcus* (n=19) |
| NucleoSpin | 28 | 0 | 218 | 0 | 246 | *Bacillus* (n=100), *Flavobacterium* (n=85), *Janthinobacterium*(n= 19), *Haemophilus* (n=10) |
