# Supplementary material for: DNA extraction protocol impacts ocular surface microbiome profile
Source: Front Microbiol. 2023 Apr 20;14:1128917. doi: 10.3389/fmicb.2023.1128917 (PMC10157640; doi:10.3389/fmicb.2023.1128917)
Supplement: SUPPLEMENTARY 4 DATA SHEET 2 — DNA concentration per protocol with and without negative controls and blank, before and after concentration (ng/μL). [file Data_Sheet_1.PDF]

	Mediaan	Q1 - Q3	n
RNeasy with control and blank	2,07	0,69 - 3,76	2
RNeasy without control and blank	2,44	1,29 - 4.08	
RNeasy after upconcentration; with control and blank	2,07	1,5 - 3,76	
RNeasy after upconcentration; without control and blank	2,44	1,85 - 4,08	
FastDNA with control and blank	0,05	0,00 - 0,52	10
FastDNA without control and blank	0,11	0,00 - 0,58	
FastDNA after upconcentration; with control and blank	1,18	0,68 - 1,72	
FastDNAafter upconcentration; without control and blank	1,41	0,81 - 1,74	
Blood & Tissue first elution with control and blank	0,04	0,00 - 0,13	14
Blood & Tissue first elution without control and blank	0,06	0,01 - 0,14	
Blood & Tissue second elution with control and blank	0,00	0,00 - 0,00	
Blood & Tissue second elution without control and blank	0,00	0,00 - 0,01	
Blood & Tissue after upconcentration; with control and blank	0,22	0,08 - 0,60	
Blood & Tissue after upconcentration; without control and blank	0,36	0,12 - 0,63	
QIAamp with control and blank	0,00	0,00 - 0,00	14
QIAamp without control and blank	0,00	0,00 - 0,00	
QIAamp after upconcentration; with control and blank	0,01	0,00 - 0,02	
QIAamp after upconcentration; without control and blank	0,01	0,00 - 0,02	
NucleoSpin with control and blank	0,93	0,60 - 1,54	1
NucleoSpin without control and blank	1,09	0,66 - 1,55	
NucleoSpin after upconcentration; with control and blank	0,93	0,60 - 1,54	
NucleoSpin after upconcentration; without control and blank	1,09	0,66 - 1,55	
PowerSoil with control and blank	2,46	1,20 - 4,35	0
PowerSoil without control and blank	3,25	1,80 - 4,56	
PowerSoil after upconcentration; with control and blank	2,46	1,20 - 4,35	
PowerSoil after upconcentration; without control and blank	3,25	1,80 - 4,56	
HostZERO with control and blank	0,01	0,00 - 0,03	0
HostZERO without control and blank	0,01	0,00 - 0,03	
HostZERO after upconcentration; with control and blank	0,01	0,00 - 0,03	
HostZERO after upconcentration; without control and blank	0,01	0,00 - 0,03	

	RNeasy	FastDNA	Blood & Tissue	NucleoSpin	PowerSoil	HostZERO	QIAamp
**Median amount of microbial DNA (ng/µl) per sample**	2,4400	0,1075	0,0559	1,0850	3,2450	0,0064	-0,0001
**Pricing**	€€€(€)	€€€	€€	€	€€	€€€€	€€€€
**Time efficiency**	++	++	+	+++	+	++	+++
**Diversity**	+	+/-	++	-	+	NA	NA
